# A Systematic Review of Alternative Artemisinin Production Strategies

**DOI:** 10.3390/ijms262412095

**Published:** 2025-12-16

**Authors:** Masoumeh Zeinali, Mohammad Sabzehzari, Didier Ménard

**Affiliations:** 1 Division of Biotechnology, Department of Agronomy and Plant Breeding, Faculty of Agricultural, University of Mohaghegh Ardabili, Ardabil 5611911367, Iran; masoume.zeinali1@gmail.com; 2 Division of Biotechnology, Department of Agronomy and Plant Breeding, College of Agriculture and Natural Resources, University of Tehran, Tehran 1417614411, Iran; 3 Malaria Genetics and Resistance Team (MEGATEAM), UR 3073—Pathogens Host Arthropods Vectors Interactions Unit, Université de Strasbourg, F-67000 Strasbourg, France; 4 Malaria Parasite Biology and Vaccines, Institut Pasteur, Université Paris Cité, F-75015 Paris, France; 5 Laboratory of Parasitology and Medical Mycology, CHU Strasbourg, F-67000 Strasbourg, France; 6 Institut Universitaire de France (IUF), F-75231 Paris, France

**Keywords:** *Artemisia annua*, antimalarial therapy, biosynthetic pathways, artificial intelligence, malaria, medicinal plants

## Abstract

Artemisinin (ART) production faces bottlenecks due to low and variable yields from its natural source, *Artemisia annua*. This limitation, coupled with expanding therapeutic potential beyond malaria, highlights the need for innovative production solutions. This systematic review aims to synthesize the evidence on alternative production platforms for ART. We searched PubMed, Scopus, Web of Science, and Google Scholar for studies published primarily between 2020 and 2025. Some search terms included “Artemisinin”, “*Artemisia annua*”, “biosynthesis”, “in vitro culture”, and “artificial intelligence”. We included primary research articles reporting on strategies for ART production. We narratively synthesized data by production theme. Our review of 30 studies identified four frontiers for ART production: (1) Enhancement in *A. annua* ART content; (2) In vitro platforms focusing on callus and cell suspension cultures, which offer precise control but face scale-up bottlenecks; (3) Heterologous expression in non-*Artemisia* plants; and (4) Scalable semi-synthetic routes using microbially fermented precursors and chemical conversion. Furthermore, the review highlights the emerging role of AI-driven predictive modeling in source discovery and process optimization. By integrating these innovations, a robust roadmap exists for sustainable ART production.

## 1. Introduction

Artemisinin (ART) and its derivatives were first isolated from *Artemisia annua* L. in the 1970s [1]. They are now indispensable in combating malaria and are at the forefront of global malaria control strategies [2,3]. Recognized for their efficacy against chloroquine-resistant *Plasmodium falciparum* parasites, ART-based Combination Therapies (ACTs) have become a cornerstone of global efforts to control and eliminate malaria [4]. More recently, the WHO has endorsed RTS, S, and R21 malaria vaccines, particularly in Africa and other regions with moderate to high malaria transmission. Although these vaccines along with ACTs have strengthened the global response, this life-threatening disease remains a significant health challenge [3]. About 3.3 billion people are at risk, with over 100 countries experiencing ongoing malaria transmission. This widespread burden has driven the market for ACTs, valued at USD 600 million in 2023, with projections indicating a compound annual growth rate of 8% from 2024 to 2030 [5].

Despite advances in ART research [6], production remains a major challenge, both in natural and synthetic contexts [7]. ART yield from *A. annua* cultivation is inherently low [8], accounting for only 0.01% to 1.5% of the plant’s dry weight [9]. Moreover, the molecule’s structural complexity makes chemical synthesis inefficient and economically unviable [10]. Collectively, these challenges highlight the critical need for robust biotechnological innovations to secure a stable ART supply [11], moving production beyond the limitations of the plant itself.

While several reviews have summarized progress in ART production [12,13,14], a systematic synthesis of the evidence across all alternative platforms is lacking. To address this gap, we performed this systematic review to answer the following research questions: 1. What are the primary biotechnological strategies currently being explored for ART production outside of conventional *A. annua* cultivation? 2. What is the reported efficacy, scalability, and commercial viability of each strategy? 3. What is the emerging role of artificial intelligence in optimizing these production systems? By systematically collating and synthesizing the available literature, this analysis identifies four key production frontiers, including enhancement in *A. annua* ART content; in vitro callus and suspension cultures; heterologous expression in non-*Artemisia* plants; and semi-synthetic routes. Overall, this review provides a comprehensive evidence base to guide future research, funding, and industrial strategy for sustainable ART production.

## 2. Methods

We conducted and reported this systematic review in accordance with the Preferred Reporting Items for Systematic Reviews and Meta-Analyses (PRISMA) guidelines [15].

### 2.1. Information Sources and Search Strategy

We performed a comprehensive literature search across the following electronic databases: PubMed, Scopus, Web of Science, and Google Scholar. The search strategy combined keywords and subject headings related to ART and various production methods.

The search string for Google Scholar is as follows: (“artemisinin” OR “artemisinic acid” OR “*Artemisia annua*”) AND (“biosynthesis” OR “metabolic engineering” OR “heterologous expression” OR “synthetic biology” OR “in vitro culture” OR “semi-synthetic” OR “bioreactor” OR “endophyte” OR “artificial intelligence” OR “machine learning”).

The search string for PubMed is as follows: (“artemisinin” [Title/Abstract] OR “artemisinic acid” [Title/Abstract] OR “*Artemisia annua*” [Title/Abstract]) AND (“biosynthesis” [Title/Abstract] OR “metabolic engineering” [Title/Abstract] OR “heterologous expression” [Title/Abstract] OR “synthetic biology” [Title/Abstract] OR “in vitro culture” [Title/Abstract] OR “semi-synthetic” [Title/Abstract] OR “bioreactor” [Title/Abstract] OR “endophyte” [Title/Abstract] OR “artificial intelligence” [Title/Abstract] OR “machine learning” [Title/Abstract]).

The search string for Scopus is as follows: (TITLE-ABS (“artemisinin” OR “artemisinic acid” OR “*Artemisia annua*”)) AND (TITLE-ABS (“biosynthesis” OR “metabolic engineering” OR “heterologous expression” OR “synthetic biology” OR “in vitro culture” OR “semi-synthetic” OR “bioreactor” OR “endophyte” OR “artificial intelligence” OR “machine learning”)).

The search string for Web of Science is as follows: TS = ((artemisinin* OR “artemisinic acid” OR “*Artemisia annua*”) AND (biosynthesis* OR “metabolic engineering” OR “heterologous expression” OR “synthetic biology” OR “in vitro culture” OR semi-synthetic* OR bioreactor* OR endophyt* OR “artificial intelligence” OR “machine learning”)).

### 2.2. Eligibility Criteria

Included studies met the following criteria: (1) Publication Type: Peer-reviewed original research articles. (2) Content: Studies developing of testing a method for producing ART or its direct precursors (e.g., artemisinic acid) using biotechnological, semi-synthetic, or synthetic methods. This included metabolic engineering, heterologous expression, in vitro culture, microbial fermentation, and chemical synthesis. (3) Language: Publications were restricted to English. (4) Timeframe: Studies published from January 2020 to October 2025 were included to capture the evolution of the field from field cultivation to synthetic biology. We excluded reviews, patents, editorials, opinion pieces, conference abstracts, and studies with insufficient data.

### 2.3. Study Selection

**Figure 1:**
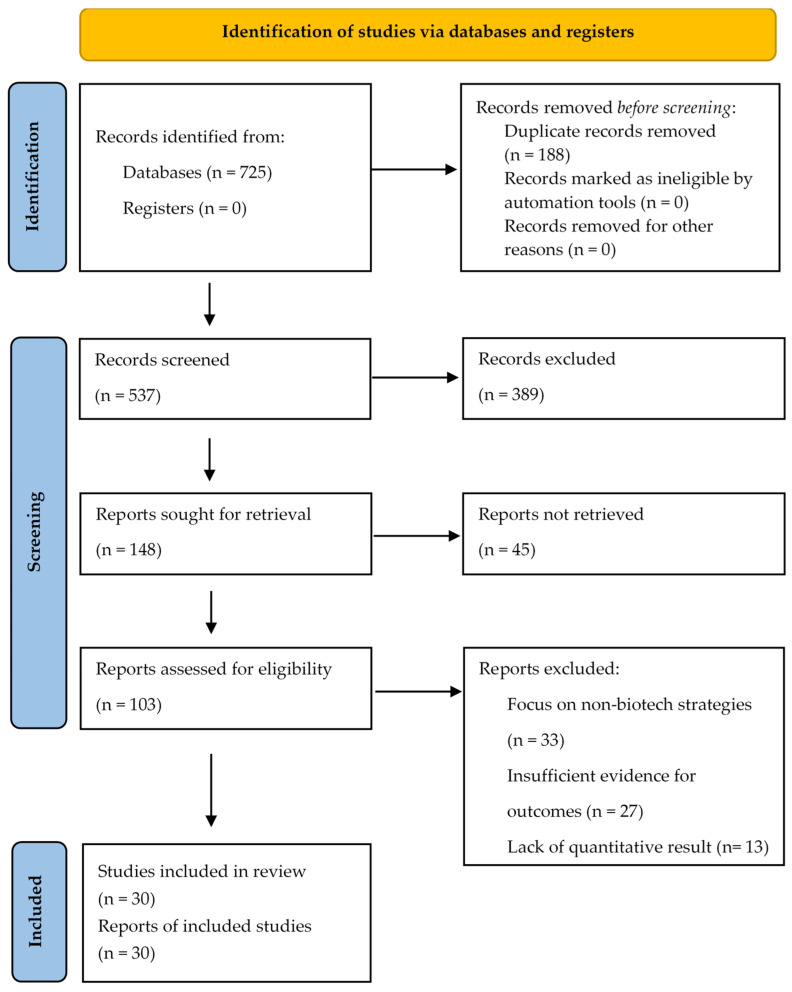
The PRISMA flow diagram illustrating the selection process.2.4. Data Extraction and Synthesis.

We imported all retrieved citations into the reference manager EndNote, and removed duplicates. Two reviewers (M.Z., M.S.) independently screened titles and abstracts against the eligibility criteria. Full texts of potentially relevant articles were then retrieved and assessed for final inclusion by the same two reviewers. Any disagreements were resolved through discussion or by consulting a third reviewer (D.M.) (Figure 1).

We used a standardized data extraction form to collect relevant information from each included study, including: production platform, author, year, brief method, outcomes (e.g., yield), key findings, limitations, and study quality assessment. Due to the heterogeneity of the study designs and reported outcomes, a meta-analysis was not feasible. Therefore, we narratively synthesized findings, organized thematically based on the identified production frontiers. To assess the quality of the included literature, we performed a qualitative evaluation of experimental rigor based on three key criteria: the presence of appropriate negative/positive controls, the use of biological replicates (*n* ≥ 3) and the application of statistical analysis to verify yield differences.

## 3. Results

### 3.1. Included Studies

The initial database search yielded 725 records. After removing 188 duplicates, we screened 537 records based on title and abstract, of which 389 were excluded. We evaluated the full texts of the remaining 103 articles for eligibility. A total of 30 articles were ultimately included in the narrative synthesis after excluding studies focused on non-biotech strategies or lacking quantitative data. The PRISMA diagram is available in Figure 1.

### 3.2. Quality Assessment of Included Studies

A qualitative assessment of the 30 included studies revealed a generally high level of experimental rigor. The majority of studies utilized appropriate controls (e.g., wild-type *A. annua* or empty-vector controls in metabolic engineering), biological replicates, and statistical methods. However, Table 1 highlights that one study was of moderate quality due to lacks a distinct positive control group [16].

### 3.3. Synthesis of Findings

**Figure 2:**
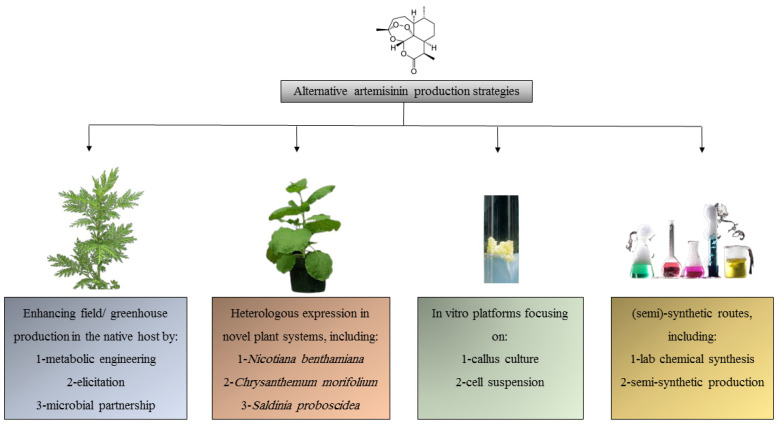
Alternative artemisinin production strategies.

The included studies reveal a landscape of innovation focused on moving ART production beyond agricultural constraints. According to Table 1, the research focus was as follows: ART enhancement in *A. annua* (*n* = 18, 60%), In vitro cultures (*n* = 6, 20%), Heterologous expression in non-*Artemisia* species (*n* = 4, 13%), and Semi-synthetic routes (*n* = 2, 7%). Noting one study overlapped categories, we synthesized the findings into four interconnected themes reflecting the major research frontiers (Figure 2).

### 3.4. Theme 1: Enhancing Production in the Native Host, Artemisia *sp.*

#### 3.4.1. Decoding the ART Biosynthetic Pathway: A Prerequisite for ART Content Enhancement

**Figure 3:**
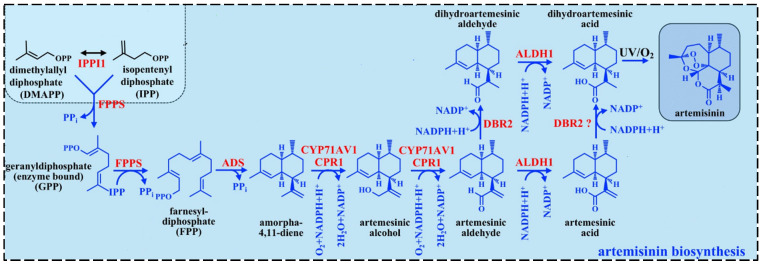
The ART biosynthetic pathway in *A. annua*. The FPP, which is originates from the activity of the mevalonate (MVA) and the methylerythritol phosphate (MEP) pathways, generates the sesquiterpene backbone, amorpha-4,11-diene. This intermediate undergoes enzymatic oxidation, mediated by cytochrome P450 monooxygenases and other redox enzymes, to yield DHAA. Finally, ART is synthesized via a sequence of enzyme-catalyzed reactions followed by non-enzymatic oxidative modifications, including light-dependent peroxidation. Abbreviations: FPP, farnesyl diphosphate; ALDH1, aldehyde dehydrogenase 1; DBR2, double bond reductase 2; CYP, cytochrome P 450 CYP71AV1; ADS, amorpha-4,11-diene synthase.

Much of the literature focuses on understanding the ART biosynthetic pathway and its regulation, which are fundamental to all engineering efforts. Understanding this intricate pathway is vital to enhancing ART production, whether by optimizing the native plant system or transferring the ART production machinery to alternative hosts. A tightly regulated cascade of enzymatic reactions and genetic controls orchestrates ART biosynthesis. Central to this process is the amorpha-4,11-diene synthase (ADS), which catalyzes the cyclization of farnesyl diphosphate (FPP) into amorpha-4,11-diene as the essential backbone of ART. This initial step sets the stage for a series of oxidative modifications catalyzed by cytochrome P450 monooxygenase (CYP71AV1) and its redox partner cytochrome P450 reductase (CPR), which sequentially hydroxylate and oxidize amorpha-4,11-diene to yield artemisinic alcohol, aldehyde, and ultimately artemisinic acid [1] (Figure 3).

A pivotal branch in the pathway is governed by artemisinic aldehyde Δ11 (13) reductase (DBR2), which redirects artemisinic aldehyde toward dihydroartemisinic aldehyde, the precursor for dihydroartemisinic acid (DHAA). Under oxidative conditions, DHAA undergoes non-enzymatic conversion to ART, a reaction finely tuned by the plant’s glandular trichome microenvironment. Another key enzyme, ALDH1, oxidizes DHAA to dihydroartemisinic acid [1].

The pathway’s regulation is a symphony of genetic interplay. Transcription factors such as WRKY, MYB, and AP2/ERF families act as molecular conductors, binding to promoters of key genes (*ADS*, *CYP71AV1*, and *DBR2*) to amplify expression under stimuli signaling [12]. Conversely, microRNAs like miR858 and miR159 fine-tune this orchestra, silencing competing pathways to channel metabolic flux toward ART biosynthesis [45]. By dissecting these molecular intricacies, researchers are not only decoding nature’s blueprint but also reengineering it via metabolic engineering and synthetic biology to increase ART yields. The discussion of ART genetic modifications is beyond the scope of this article, so readers can refer to relevant reviews [46].

#### 3.4.2. Artemisia Species as Native Hosts

Evidence from multiple studies indicates that while the ultimate goal may be fully ex planta production, significant biotechnological innovations focus on enhancing ART synthesis within plant systems themselves (Table 1). *Artemisia* plants are the primary natural source of ART and the potential target for engineering. The ART biosynthesis pathway is thought to be an ancestral trait shared by plants within the genus *Artemisia* [47]. This theory is partially supported by studies reporting the expression of ART biosynthesis-related genes in several *Artemisia* species, including *A. vulgaris*, *A. absinthium* and *A. verlotiorum* [48]. Recent evidence has further strengthened this hypothesis with the detection of ART intermediates in *A. alba* for the first time [49]. Interestingly, ART accumulation does not peak at the same growth stage in all *Artemisia* species. In *A. absinthium*, ART levels are highest during flowering, whereas in *A. vulgaris* and *A. annua*, the peak occurs during the budding stage [1]. This variability explains why some studies fail to detect ART or report it at undetectable levels, as these investigations often focus on specific growth stages when ART synthesis is minimal.

#### 3.4.3. ART Content Enhancement by Metabolic Engineering

Table 1 identified transcription factor (TF)/key enzyme overexpression technique as the most used genetic engineering approach. For example, Yuan et al. [17] engineered *A. annua* via overexpression of the TF MYC3, resulting in a 1.4 to 1.8-fold increase in ART content. Similarly, Hu et al. [18] overexpressed PDF2, increasing GST density by up to 82% and ART content by 55–73%. Other related studies focused on transcription factors such as YABBY5 [50], MYB108 [51], and ABI5 [24]. Notably, Li et al. [16] employed a synthetic biology multigene construct to reconstruct the ART pathway, achieving ART content up to 24.7 mg g^−1^ DW (a 2.4 to 3.4-fold increase compared to the control). However, limitations persist; for example, Yuan et al. [17] noted that yield increases from MYC3 were less than those from ART-specific regulators, suggesting internal metabolic feedback limits.

The unstable and low accumulation of ART in *A. annua* (1–10 mg g^−1^ dry weight) results primarily from its biosynthesis, which occurs almost exclusively in the glandular secretory trichomes (GSTs) located on the leaf and flower epidermis. These specialized 10-cell structures are highly fragile and are often lost from *A. annua* tissues during harvest, posing a main challenge for traditional field-based approaches [9]. In addition to their role in ART production, GSTs also regulate the biosynthetic pathway through various mechanisms. For example, several transcription factors and GST developmental transporters have been identified as critical modulators of ART accumulation. One such modulator is the transporter ABCG40, which its overexpression in GSTs increases ART levels [31]. Transcription factors such as WRKY and MYB also influence ART biosynthesis through complex regulatory mechanisms [22,51]. Therefore, regulatory networks are crucial in controlling GST-localized ART biosynthesis [52,53]. ART biosynthetic genes also show different expression patterns depending on the developmental stage of the GSTs. In young leaves, GSTs prominently express ART synthesis genes, whereas in older leaves this expression is almost negligible [54]. This observation highlights that efficient ART biosynthesis is predominantly associated with immature GSTs [16].

Until recently, GSTs were believed to be the sole cellular structures capable of producing ART. This assumption remained unchallenged until researchers discovered that non-GST cells also possess the ability to synthesize ART [38]. Emerging evidence indicates that GST and non-GST cells utilize distinct mechanisms in the final steps of ART synthesis. In GSTs, ART is formed through the autoxidation of DHAA, whereas in non-GST cells, enzymatic reactions mediate the conversion of DHAA to ART [9]. This discovery opens new avenues for manipulating ART production in different cell types or potentially in non-specialized cells in culture.

#### 3.4.4. Elicitation Strategies in Field/Greenhouse Systems

Elicitation strategies were prominent in the reviewed studies. Tripathi et al. [19] demonstrated that co-exposure of 0.5 mg L^−1^ AgNPs and 3 h of UV-B irradiation resulted in a ~4.27-fold increase in ART content, though higher concentrations (5.0 mg L^−1^) proved toxic. Foliar elicitation with Menadione Sodium Bisulphite (MSB) increased ART by 62.37% [20]. Soil application of Graphene increased GST density by 30–80% and ART content by 5% per unit weight, though high concentrations (>100 mg/L) were toxic [21]. Phytohormones also played a key role; exogenous Indole-3-acetic acid (IAA) increased ART 1.9-fold [25], while Strigolactone increased GST density by 21% [27]. Under stress conditions, Nitric Oxide (NO) enhanced ART under cadmium stress [26], and ABA enhanced ART under Copper stress [30].

##### Elicitation by Phytohormones

A major advantage of field/greenhouse platforms is the ability to apply phytohormone elicitors to boost ART biosynthesis. Phytohormones, such as abscisic acid (ABA), gibberellins (GA), jasmonic acid (JA), MeJA, indole-3-acetic acid (IAA), and salicylic acid are potent modulators of secondary metabolite biosynthesis in *A. annua* (Table 1). These compounds enhance ART accumulation by upregulating genes and transcription factors associated with its biosynthetic pathway [46] when applied under controlled in vitro conditions.

Jasmonic Acid (JA) induces the expression of key transcription factors, including SPL2, TCP15/14, ERF1/2, WRKY17/9, GSW1, bHLH113, and MYC2 [55]. Transcriptomic studies have also identified genes such as *TUBBY*, *Ulp1*, *AATL1*, *BAMT*, and *ORA* as critical hubs in the MeJA-induced ART biosynthetic network [56,57]. Abscisic acid (ABA) signaling involves the transcription factor bZIP1. When phosphorylated by the ABA-responsive kinase APK1, bZIP1 forms a complex with bH113 and GSW1. This complex positively regulates ART biosynthesis [16]. ABA also plays a role in the GST development and reactive oxygen species homeostasis [30]. Indole-3-acetic acid (IAA) increases ART content by upregulating the expression of *DBR2*, *ALDH1*, *CYP71AV1*, and *ADS*. Additionally, IAA enhances GST density, although the precise mechanisms underlying these effects remain unclear [25]. Gibberellins (GAs) regulate GST development and ART production by increasing trichome density and the transcript levels of *CYP71AV1*, *ADS*, and *FPS* [53]. Salicylic acid (SA) also induces ART biosynthesis by activating transcriptional factors such as WRKY17 and the TGA6-NAC1 complex, which upregulate the expression of *ERF1* and other ART biosynthetic genes [29].

##### Elicitation by Stressful Factors

Moderate levels of environmental stress can act as elicitors, enhancing secondary metabolite production in controlled in vitro environments. Heavy elements, for instance, promote ART accumulation, potentially through non-enzymatic conversion of ART precursors under oxidative stress [26,30]. While the mechanisms underlying these effects remain unclear, oxidative stress appears to play a significant role. The variability in stress responses across studies highlights the need for standardized experimental conditions (Table 1). Combining multiple stressors in a controlled manner may activate synergistic signaling pathways, further enhancing ART production.

##### Elicitation by Microbial Partnerships

Strategies using microbial consortia showed promise. Endophytes are plant-associated microorganisms that inhabit internal plant tissues without causing harm. Their presence often confers numerous benefits to the host plant, including increased tolerance to stressful conditions, growth, and secondary metabolite production [58]. Notably, *A. annua* endophytes have been shown to synergistically increase both biomass and ART accumulation. When a combination of endophytes—including *Acinetobacter pittii*, *Burkholderia* sp., *Bacillus subtilis*, and *B. licheniformis*—was applied as a bio-inoculant, ART yields surpassed those achieved with any of these bacteria in monocultures [33]. Recently, it has been found that a plant growth-promoting endophytic consortium including *B. subtilis*, *B. licheniformis*, *Burkholderia* sp., and *Acinetobacter pittii* improved ART biosynthesis via up-regulating *ISPH*, *SQC*, *ADH1&2*, *HMGS*, *HMGR*, *DXR1*, *DXS1*, *FPS*, *ADS*, and *CYP7AV1* [32]. These findings suggest that employing an endophytic consortium offers a promising and sustainable biotechnological strategy to enhance ART production. This approach not only increases ART yield but also aligns with eco-friendly and cost-effective agricultural practices [59].

### 3.5. Theme 2: ART Production by In Vitro Systems

Plants exhibit rapid responses to even minor changes in their chemical and physical environments. This characteristic makes tissue and cell culture an effective platform for controlling environmental factors to optimize the production of secondary metabolites [60,61,62]. Achieving this requires two critical steps: first, the cultivation of plant cells or tissues in vitro, and second, the application of effective elicitors using precise protocols to enhance the production of the target metabolite.

Table 1 primarily highlights callus and cell suspension in the included timeframe. Nabi et al. [59] achieved a maximum ART yield of 780 ng g^−1^ DW in *A. maritima* callus cultures using MeJA elicitation. Mosoh and Vendram [60] utilized in vitro callus culture with PGRs and AgNO_3_, but noted that ART content remained lower than in control plants. Lim et al. [61] explored in vitro cell suspension cultures with LED lighting, identifying a trade-off where optimal biomass conditions were suboptimal for ART accumulation. Other in vitro approaches included foliar application of Iron Oxide Nanoparticles [62] and cultivation under monochromatic blue light, which upregulated *ADS* expression [27].

Light also plays a critical role in regulating ART biosynthesis by modulating GST density, allowing precise control over this factor. The COP1-HY5-BBX21 module is central to this light-mediated control. In darkness, COP1 interacts with BBX21 and AaHY5, targeting them for degradation via the ubiquitin-26S proteasome pathway, which suppresses ART biosynthesis. Conversely, light inhibits COP1 activity, allowing HY5 and BBX21 to accumulate. HY5, either independently or as part of the HY5-BBX21 complex, activates genes such as *MYB108*, *ORA*, and *GSW1*, driving ART biosynthesis and GST development [63]. Different light spectra also influence ART production in specific ways when applied to cultures: red light stimulates the mevalonate (MVA) pathway; yellow light increases secretory trichome density; green light enhances volatile compound variations; and blue light thickens the leaf epidermis, increases cell size, promotes *ADS* expression, and boosting ART levels [38]. Therefore, optimizing light conditions is a key strategy in vitro.

### 3.6. Theme 3: Heterologous Expression in Novel Plant Systems

Table 1 lists four key studies in this domain. Guo et al. [40] reported transient expression in *Nicotiana benthamiana*, producing DHAA (0.51 mg g^−1^ DW) and ART (0.041 mg g^−1^ DW) after UV treatment. Firsov et al. [43] explored *Chrysanthemum morifolium* with *A. annua* genes, but notably, ART was not detected via GC-MS, highlighting the biochemical limits of the host. In a separate study, Firsov et al. [41] successfully transferred the entire biochemical pathway into *Chrysanthemum*, but transformation efficiency was low (0.33%). Additionally, Randrianarivo et al. [42] isolated a natural stereoisomer, (−)-6-epi-artemisinin, from *Saldinia proboscidea*, though yields were low (2.5 mg from 500 g DW).

**Figure 4:**
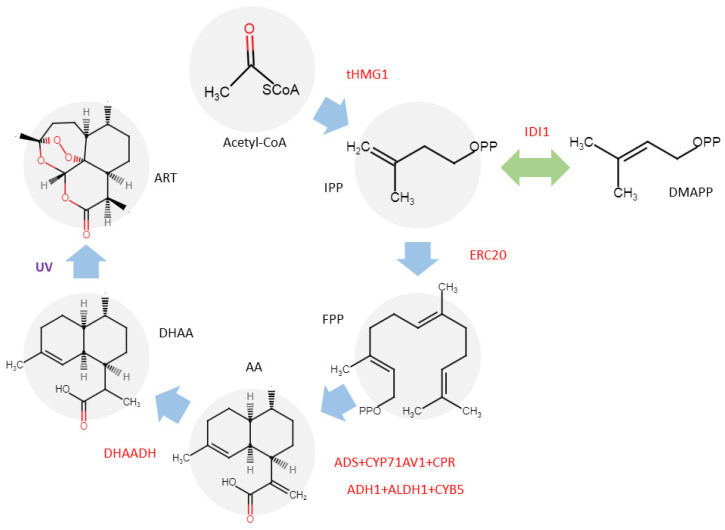
Reconstruction ART biosynthesis pathway in *N. benthamiana*. The transferred genes are shown in red [40].

A significant step ‘beyond the *Artemisia’* involves transferring the ART biosynthetic pathway into alternative hosts. The biochemical evaluation of *Saldinia proboscidea* has led to the discovery of an isomer of ART, specifically (−)-6-epi-artemisinin [42]. This finding represents the inaugural report of an ART-related compound isolated from a non-*Artemisia* genus, signifying a pivotal milestone in the expansion of potential ART sources beyond the traditional genus. Subsequently, *Chrysanthemum morifolium* has attracted the attention of biotechnologists due to its high content of sesquiterpenoids, terpenoids, and their precursors. This species has been successfully transformed with key genes from the ART biosynthetic pathway, including the *HMGR* gene from yeast and the *DBR2*, *CPR*, *CYP71AV1*, and *ADS* genes from *A. annua* [41,43]. Recently, researchers successfully reconstructed the ART biosynthetic pathway in *Nicotiana benthamiana* by transiently expressing eight proteins alongside the targeted optimized enzyme DHAADH. While this system enabled the production of the precursor DHAA at a concentration of 0.5 mg g^−1^ DW, ART was not initially detected within the plant tissue. To overcome this, the researchers irradiated the *N. benthamiana* leaves with UV light to accelerate the specific auto-oxidation of DHAA, ultimately achieving an ART content of 0.04 mg g^−1^ DW (Figure 4) [40]. These advancements highlight the feasibility of transplanting the ART biosynthetic pathway into non-*Artemisia* plants, offering a promising avenue for diversifying and enhancing ART production in alternative plant chassis.

### 3.7. Theme 4: Chemical and Semi-Synthetic Frontiers

**Figure 5:**
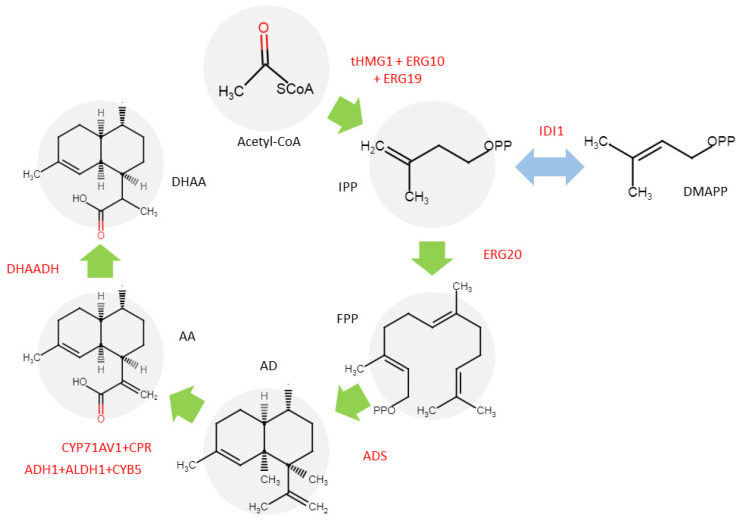
Synthetic biology of ART biosynthesis pathway in *S. cerevisiae*. The transferred genes are shown in red [40].

Table 1 includes two studies representing this frontier. Guo et al. [40] described a metabolic engineering approach in *S. cerevisiae* using a bioreactor, producing 3.97 g L^−1^ of DHAA (Figure 5). Moreover, Chen et al. [44] achieved a 41% yield of ART from DHAA using “Dark” Singlet Oxygen, providing a scalable method. However, limitations were noted, such as long reaction times for chemical conversion.

The included literature covers purely chemical and hybrid biochemical routes. Research into the chemistry of ART expanded rapidly between 1985 and 2015. While the total synthesis of ART has been achieved, the evidence consistently shows that it remains commercially impractical due to the molecule’s complexity and high costs. Consequently, research has pivoted to semi-synthesis, where genetically engineered yeast produce artemisinic acid, which is then chemically converted to ART. This method is a key ‘beyond the plant’ innovation that provides a scalable alternative source [40].

The first semi-synthesis of ART was successfully reported by Chen et al. [44]. This method, which involves the conversion of artemisinic acid to ART, is technically straightforward, scalable and eliminates the need for intermediate purification. By using singlet oxygen resources, the process also avoids the need for specialized photochemical tools. Semi-synthesis represents a practical approach to ART production that decouples the final steps from the plant source. However, further research is essential to optimize this method and achieve a cost-effective solution for large-scale applications [64].

As a commercial alternative, biochemical processes have been developed to produce ART on an industrial scale. One such approach involves genetically engineered yeast-mediated fermentation to produce artemisinic acid. After conversion, artemisinic acid is then converted to ART in a semi-synthetic reaction. This method utilizes microbial cell factories, a key ‘beyond the plant’ innovation, providing a scalable alternative source of ART and has the potential to make a significant contribution to meeting global demand [65].

### 3.8. A Trend: The Role of AI in Discovery and Optimization

Artificial intelligence (AI) represents a set of tools to improve ART production beyond plant systems in future research. AI, particularly machine learning (ML) and deep learning (DL), offers a revolutionary avenue in natural product discovery within medicinal plants and microbial systems. By streamlining the analysis of vast biological datasets, ML and DL are becoming indispensable in the search for new ART sources or pathways [66]. Machine learning can effectively handle large-scale data from plant genomics, transcriptomics, metabolomics, and phenomics, identifying patterns that correlate ART production with various stimuli. Predictive models can estimate the likelihood of ART production in plants or microbes with genetic or chemical profiles similar to *A. annua*. Subtle patterns detected by ML and DL tools may reveal the existence of ART biosynthetic pathways in previously unstudied species. By analyzing environmental factors affecting ART yield, ML/DL facilitates recommendations for optimal growth conditions, ensuring maximum production in bioreactors or other controlled systems. Algorithms can also pinpoint the key features, such as genes, enzymes, and pathways linked to ART biosynthesis, aiding in the selection of promising candidate hosts or engineering targets [67]. For instance, the integration of ML frameworks with multi-omics data has shown promise in resolving complex biosynthetic pathways. Bai et al. [68] utilized the AutoGluon-Tabular framework to analyze data from Arabidopsis, successfully predicting genes encoding enzymes for three major categories of plant secondary metabolites, including terpenoids. Their analysis identified that genomic and proteomic features were the most critical predictors of model performance, surpassing transcriptomic or epigenomic data. Furthermore, the model demonstrated robust cross-species applicability, achieving high predictive accuracy when validated in poppy, grape, maize, and tomato. Applying such ML strategies to *Artemisia* could accelerate the identification of novel candidates involved in ART biosynthesis, overcoming the limitations of traditional analysis approaches. These strategies accelerate research efforts, offering time-efficient and cost-effective methods to identify new ART sources and optimize biotechnological production processes.

## 4. Discussion

This systematic review synthesizes the evidence from 30 studies on biotechnological strategies for ART production. This review directly addressed the core questions guiding the inquiry into alternative ART production. Addressing our first question regarding the primary biotechnological strategies being explored, this review identified four major frontiers: (1) Enhancement in *A. annua* ART content; (2) In vitro callus and suspension cultures; (3) Heterologous expression in plants like *Nicotiana* and *Chrysanthemum*; and (4) Semi-synthetic production combining yeast fermentation and chemical synthesis.

Answering our second question on efficacy and scalability, the evidence shows that the semi-synthetic pathway remains the most scalable alternative, though it faces challenges such as catalyst stability and reaction times [40]. Table 1 highlights some challenges in in vitro callus and suspension systems, such as trade-offs between biomass and ART accumulation [35] and lower yields compared to the field context [28]. Finally, heterologous expression in non-*Artemisia* plants is currently suited for novel plant chassis [40].

Finally, addressing our third question, the role of computational tools has evolved into a critical enabler across all frontiers; AI and machine learning are no longer just theoretical but are actively being used to mine genomic data for novel biosynthetic pathways, predict optimal hosts, and dynamically optimize growth conditions to maximize metabolic flux and final yield.

### 4.1. Strengths and Limitations of This Review

The primary strength of this review is its systematic and comprehensive methodology, which provides a transparent and reproducible synthesis of the available evidence across multiple production frontiers. However, several limitations should be noted. First, our search was restricted to English-language publications, potentially introducing language bias. Second, the heterogeneity of the methodologies prevents a quantitative meta-analysis. While our quality assessment indicates that the majority of studies utilized sufficient biological replicates and controls, the variability in extraction methods and yield reporting units (e.g., % dry weight vs. mg/L) limits direct quantitative comparison. Finally, as with any field, there is a risk of publication bias, where studies with positive or significant results are more likely to be published than those with null findings.

### 4.2. Implications for Future Research and Policy

Based on the synthesized evidence, we recommend the following directions for future research: (1) Head-to-head comparisons: There is a need for studies that directly compare the economic and environmental lifecycle costs of the leading alternative platforms. (2) AI integration: Research should focus on integrating AI not just for discovery but for real-time process control in bioreactors to maximize metabolite flux. (3) Regulatory pathways: As these technologies mature, research into the regulatory and quality control frameworks necessary for pharmaceutical-grade ART from non-traditional sources is critical. (4) For policymakers and funding bodies, our review highlights the need for continued investment in these diverse biotechnological platforms to create a resilient and stable supply chain for this essential medicine.

### 4.3. Critical Barriers to Translation

Despite the biological efficacy of these platforms, several major challenges remain. First, significant knowledge gaps persist regarding non-glandular synthesis. While the enzymatic capability of non-GST cells has been identified, the specific transport mechanisms and storage compartments preventing autotoxicity in these cells are unknown, limiting our ability to engineer high-yield non-glandular chassis. Second, regulatory challenges for non-traditional sources are distinct from natural ART. ART derived from genetically modified microbial consortia or novel plant hosts requires rigorous GMP validation to ensure that host-specific metabolites do not contaminate the final pharmaceutical product, a regulatory pathway far more complex than standard botanical extraction.

Technically, plant systems frequently encounter metabolic bottlenecks; redirecting carbon flux toward the ART pathway often compromises host fitness by depleting the common precursor FPP, which is essential for sterol biosynthesis. Furthermore, the accumulation of intermediates like artemisinic aldehyde can be cytotoxic to microbial hosts, necessitating pathway balancing and transport engineering. Economically, the barrier is equally steep. While in vitro systems offer control, the capital expenditure (CAPEX) for large-scale bioreactors and the operational costs (OPEX) for energy, media, and sterile maintenance significantly exceed the costs of open-field farming, where solar energy and soil are free inputs. Moreover, downstream processing (DSP) and purification from complex biomass remains a major cost driver, often accounting for 60–80% of total production costs. Consequently, biotechnological ART must not only match but exceed the yield efficiency of *A. annua* to compete with the fluctuating but generally low cost of natural ART.

### 4.4. Comparative Analysis of Alternative Platforms of ART Production

To systematically evaluate the commercial viability of these diverse biotechnological approaches, it is essential to contrast their performance against the current agricultural standard. Table 2 provides a comparative overview of the identified production frontiers, analyzing them based on yield potential, scalability, and Technology Readiness Level (TRL). While semi-synthetic routes via yeast fermentation have achieved industrial maturity (TRL 9) comparable to field cultivation, other platforms such as in vitro cultures and heterologous expression in non-*Artemisia* hosts currently occupy distinct developmental niches. This comparison highlights that while in vitro methods offer superior biological control, their widespread adoption is currently limited by the economic constraints compared to the established efficacy of field cropping.

Building on these findings, we propose a criteria-based framework for selecting the optimal production platform based on specific end-use requirements and resource constraints. Evidence suggests that semi-synthetic production via yeast fermentation remains the superior choice for industrial-scale pharmaceutical supply chains requiring high purity, consistency, and total independence from climatic variables. In contrast, for sustainable agricultural improvement in regions with existing cultivation infrastructure, the application of microbial consortia provides a cost-effective, eco-friendly strategy to enhance biomass and ART content without the regulatory complexities of genetic modification. In vitro cultures emerge as the preferred platform for controlled laboratory production and metabolic engineering. Finally, heterologous expression in non-*Artemisia* plants is currently best suited for experimental pathway elucidation and exploring novel biological chassis.

## 5. Conclusions

The vital need for a scalable supply of ART, hindered by the inherent limitations of *A. annua* cultivation, necessitates a paradigm shift towards biotechnological production systems. Beyond scientific feasibility, these strategies offer industrial and socioeconomic benefits. Industrially, moving production from the field to controlled bioreactors (via semi-synthesis) ensures a year-round supply chain that is resilient to climate variability. While in vitro cultures show potential, optimization of trade-offs between growth and secondary metabolism is still required. Economically, AI-driven process optimization promises to lower production costs, thereby stabilizing the historically volatile market prices of ACTs. Socially, securing a consistent supply of pharmaceutical-grade ART is an ethical imperative; it ensures equitable access to life-saving therapies in high-burden regions, directly supporting the WHO’s malaria elimination goals. By integrating these innovations, researchers can chart a robust roadmap toward sustainable ART production, reshaping it from a variable botanical extract into a reliable cornerstone of global health.

## Figures and Tables

**Table 1:** Characteristics of the included studies along with their quality assessment. Abbreviation: RQ *, research quality; TF, transcription factor.

Platform	Authors	Year	Breif Method	Outcomes	Key Findings	Limitations	RQ *
*A. annua*	Yuan et al., [17]	2025	Genetic engineering of *A. annua* via overexpression of the TF MYC3.	- ART content increased by 1.4 to 1.8 fold compared to the control. - GST density increased by 1.4 to 1.5 fold vs. the control. - DHAA content increased 1.2–1.65 fold vs. the control	- MYC3 is a JA-induced bHLH TF that regulates GST development and ART biosynthesis. - It directly activates key ART genes. - It interacts with HLH1 and bHLH113 as a co-activator to enhance transcription of *ADS* and *DBR2*, completing the ART pathway regulation.	The yield increase from overexpressing MYC3 was less than from overexpressing ART-specific regulators, suggesting internal metabolic feedback limits extreme accumulation.	High
*A. annua*	Hu et al., [18]	2025	Genetic engineering: overexpression of *PDF2* in *A. annua*	GST density increased by up to 82%; ART content increased by 55–73% compared to the control.	PDF2 promotes GST formation and ART biosynthesis. It is induced by JA and acts downstream of HD1, regulating *GSW2* expression while interacting with the repressor MYB5.	Multi-gene coordination was required for effective GST enhancement.	High
*A. annua*	Tripathi et al., [19]	2024	Co-exposure of 0.5 mg L^−1^ AgNPs and 3 h of UV-B irradiation	~4.27-fold increase in ART content compared to the control.	The combined treatment upregulated key biosynthetic genes (*ADS* and *CYP71A1*) and increased the density of GSTs.	Higher concentrations of AgNPs (5.0 mg L^−1^) decreased ART by ~1.87-fold compared to the control.	High
*A. annua*	García et al., [20]	2024	Foliar Elicitation with Menadione Sodium Bisulphite (MSB) (100 mL)	Amount: ART 3.71 mg g^−1^ DW; Increase: 62% increase over the control (with 1 mM MSB).	MSB up-regulated key early biosynthetic pathway genes: *HMGR* and *DXS*.	MSB higher than 1 mM caused necrosis, which reduced ART compared to the optimal dose.	High
*A. annua*	Cao et al., [21]	2024	Soil application of Graphene	- ART content increased by 5% per unit weight compared to the control. - Density of GSTs increased by 30–80%.	- Graphene exposure increased H_2_O_2_ levels (~60%), which inhibited *Dicer* gene expression. - This suppressed miR828 biogenesis, leading to the upregulation of its target gene *MYB17*, a positive regulator of GST initiation and ART biosynthesis.	While 10–20 mg L^−1^ promoted growth, 100 mg L^−1^ was toxic.	High
*A. annua*	Kayani et al., [22]	2023	Genetic Engineering & Elicitation: Overexpression of TF YABBY5, co-expression with anti-AaJAZ8, and MeJA.	- Transgenic YABBY5- plants treated with MeJA showed the highest ART (~26 mg g^−1^). - The combined strategy resulted in a 2-fold increase compared to control. Conversely, YABBY5 antisense reduced ART (~10 mg g^−1^ DW).	- YABBY5 interacts with WRKY9 to activate the promoters of *GSW1* and *DBR2*. - GSW1 was found to be an upstream activator of YABBY5, creating a self-reinforcing loop. - JAZ8 interacts with YABBY5, inhibiting its activity.	- In the absence of stress signals, potent TFs (such as YABBY5) are bound and inactivated by repressor proteins (JAZ8), limiting the plant’s biosynthetic capacity.	High
*A. annua*	Liu et al., [23]	2023	Metabolic Engineering: Overexpression of the TF MYB108 in *A. annua*.	ART contents in MYB108-overexpressing lines increased by 70–90% compared to the control (20–22 mg g^−1^ DW).	MYB108 acts as a node integrating light and JA signaling to positively regulate ART biosynthesis. It functions by forming a complex with the TF GSW1 to enhance *CYP71AV1* expression. Its stability is regulated by light and its activity is repressed by JAZ8 in the absence of JA.	The study was unable to utilize gene editing due to technical limitations in establishing an effective system for *A. annua* at the time; therefore, the researchers could not obtain specific knockout mutants.	High
*A. annua*	Li et al., [16]	2023a	Synthetic biology strategy: Multigene construct to promote GST formation and reconstruct the ART pathway in *A. annua*.	ART content was reached to 24.7 mg g^−1^ DW in transgenic lines. In other words, 2.4 to 3.4-fold higher than control plants.	Combined strategy of increasing GST density and enhancing biosynthetic gene expression had a cumulative effect on ART yield.	Uneven *MsGSW2* expression levels across transgenic lines, possibly due to genomic insertion position or epigenetic regulation.	Medium
*A. annua*	Li et al., [24]	2023b	Genetic engineering via overexpression of the transcription factor AaABI5 in *A. annua*.	ART content increased by ~1.7-fold in ABI5-overexpressing lines compared to the control.	- ABI5 is a integrator that connects light and ABA signaling to promote ART biosynthesis. - ABA-induced upregulation of biosynthetic genes is dependent on light. - The light-signaling TF HY5 directly binds to the *ABI5* promoter and activates its expression, placing ABI5 downstream in the light-regulation pathway.	The study identifies a key integrator but the complete signaling cascade and post-translational regulation within the light/ABA crosstalk network remain to be fully elucidated.	High
*A. annua*	Guo et al., [25]	2023b	Exogenous application of Indole-3-acetic acid (IAA)	ART content increased 1.9-fold to 1.1 mg g^−1^ DW (from the control). DHAA increased 2.1-fold to 0.51 mg g^−1^ DW.	IAA promoted plant growth, increased trichome density, and upregulated the expression of key ART biosynthetic genes (*AaADS*, *AaCYP71AV1*, *AaALDH1*, *AaDBR2*).	Long-term stability of the effect in field conditions	High
*A. annua*	Wani et al., [26]	2023a	Exogenous application of nitric oxide (NO) under cadmium stress	NO (200 μM) led to 557 µg g^−1^ DW ART (14% increase vs. the control)	NO enhanced photosynthetic efficiency, protected GST density, length, and width	The exact signaling mechanisms of NO in enhancing ART biosynthesis are not fully understood.	High
*A. annua*	Wani et al., [27]	2023b	Strigolactone (8 µM)	Increased GST density by 21%; ART content improved by 30% (7.3 mg g^−1^ DW).	Improved attributes of GSTs as the site of ART synthesis.	The mechanism linking strigolactone signaling to ART biosynthesis requires further elucidation.	High
*A. annua*	Sayed and Ahmed [28]	2022	Field cultivation with elicitation using Gamma irradiation (2.5 KGy), Nano-selenium (30 ppb), or Chitosan (250 ppm) coupled with humic acid	The combination of Chitosan and Moringa resulted in a 112% increase in ART yield compared to the control.	- A synergistic relationship was observed between elicitors and fertilizers. - Chitosan was the most effective elicitor (Chitosan > Nano-selenium > Gamma irradiation), and Moringa extract was the superior fertilizer (Moringa > Humic acid > NPK).	-	High
*A. annua*	Chen et al., [29]	2021b	Overexpression of WRKY17 in *A. annua*	ART increased by 49.5–87% in overexpression lines compared to the control	- WRKY17 directly binds to the promoter of *ADS* gene and activates its expression. - Expression of WRKY17 is induced by SA and MeJA.	-Field performance and stability of transgenic lines not assessed.	High
*A. annua*	Zehra et al., [30]	2020	Exogenous application of ABA (100 μM) under Copper (Cu) stress	Cu40 (40 mg kg^−1^) led to 417 µg/g DW ART (38% decrease vs. control) Cu40 + ABA led to 538 µg g^−1^ DW ART (29% increase vs. Cu40; still 20% lower than control)	ABA protected GST density, area, and ultrastructure. The increase in ART is linked to ABA-mediated upregulation of defense mechanisms and enhancement of biosynthetic gene expression.	The study was conducted in pot conditions with soil-applied Cu, which may not fully represent field-level complex environmental interactions.	High
*A. annua*	Fu et al., [31]	2020	Genetic Engineering: Overexpression of the pleiotropic drug resistance (PDR) transporter gene *ABCG40*.	- The ABCG40-overexpressing transgenic lines produced 1.54- to 2-fold higher ART compared to control. - Conversely, knockdown lines showed a decrease in ART (up to 17% compared to control).	- ABCG40 is a plasma membrane-localized ABA importer expressed in trichomes. - Overexpression led to higher ABA, which upregulated key ART biosynthesis genes.	While ABA was known to enhance ART, the basis of ABA transport in *A. annua* was unknown, limiting the ability to manipulate this pathway for higher yields.	High
*A. annua*	Tripathi et al., [32]	2025	Endophytic Consortium: Inoculation of *A. annua* with a consortium of endophytes: 1. *Bacillus subtilis*; 2. *B. licheniformis*; 3. *Burkholderia* sp.; 4. *Acinetobacter pittii*.	ART content increase: Under normal conditions: +51.61% Under drought stress: +32.87% Under salinity stress: +25.64% Under waterlogging: +31.57%	- The consortium up-regulated key structural genes and down-regulated SQS, diverting metabolic flux toward ART biosynthesis. - The consortium performed better than single inoculants by covering functional vulnerabilities of individual strains.	- Stresses further restrict plant growth. - As a single strain limitation, no single endophyte can up-regulate every step in the ART biosynthesis, necessitating a synergistic consortium.	High
*A. annua*	Tripathi et al., [33]	2020	Endophytic Consortia Inoculation: Application of endophytes (*Bacillus* spp., *Burkholderia* sp., and *Acinetobacter* sp.) to *A. annua*.	A consortium of four endophytes (*B. subtilis*, *B. licheniformis*, *Burkholderia* sp., *and A. pittii*) increased ART by 658% over the control, exceeding the yield of any single microbe used in isolation.	Transgressive Overyielding: While monocultures improved yields, ecological interactions in the four-strain consortium led to productivity higher than the single strain.	A number of microbial combinations resulted in “transgressive underyield,” meaning they performed worse than monocultures due to antagonistic interactions.	High
In vitro culture	Nabi et al., [34]	2025	Elicitation of callus cultures; Species: *A. maritima* Elicitor: MeJA (50–100 µM)	Maximum ART yield: 780 ng g^−1^ DW. In other words, a ~2.44-fold increase compared to the control.	The optimal condition was 100 µM MeJA. Antioxidant enzyme activities increased alongside ART, suggesting the stress drives metabolite accumulation.	*A. maritima* is an under-utilized species compared to *A. annua*, lacking protocols for scalable production.	High
In vitro culture	Mosoh and Vendram [35]	2025	In vitro callus culture elicitation with PGRs (BAP, NAA, 2,4-D) and AgNO_3_	Highest callus ART: 487 µg mL^−1^ by using 5 mg L^−1^ BAP + 1 mg L^−1^ NAA.	AgNO_3_ has a dual, context-specific role: alleviates oxidative stress under 2,4-D but exacerbates it under NAA.	Lower ART in vitro: Callus ART content remained lower than in control, highlighting a bottleneck in callus cultures.	High
In vitro culture	Lim et al., [36]	2025	In vitro cell suspension culture	- Highest ART content in the absence of KNO_3_ & KH_2_PO_4_. - Red + blue LED increased biomass; Red LED alone gave highest ART.	- Macronutrient levels create a trade-off: optimal concentrations for biomass are suboptimal for ART accumulation. - Light quality differentially regulates biomass (red + blue) and ART (red) production.	The inverse relationship between optimized biomass conditions and conditions that maximize ART content presents a key optimization challenge for maximizing yield.	High
In vitro culture	Ayoobi et al., [37]	2024	Foliar application of Iron Oxide Nanoparticles (Fe_3_O_4_-NPs) to *A. annua* in vitro	ART content increased after 96 h by: • 98% (50 mg L^−1^) •76% (100 mg L^−1^) • 77% (200 mg L^−1^) compared to the control.	Fe_3_O_4_-NPs activated an enzymatic defense system. This response stimulated the expression of key biosynthetic genes (*WRKY1*, *MYB2*, *HMGR*, *CYP71A1*), increased ART production.	Scaling the nanoparticle application for commercial field production	High
In vitro culture	Lopes et al., [38]	2020	In vitro cultivation under monochromatic blue light (LED, λmax = 475 nm)	Enhanced ART production through GST density and *ADS* expression modulation.	Blue light upregulates the expression of *ADS* gene. It promotes the plastidic DOXP/MEP pathway, enhances GST frequency.	The study was conducted under in vitro conditions; translating these findings to large-scale or field cultivation requires further validation.	High
In vitro culture	Xiao et al., [39]	2020	Exogenous application of the airborne signaling molecule β-ocimene (10 μM).	ART content reached up to 25 mg g^−1^ DW in plants treated with β-ocimene.	- Upregulation of key genes in both precursor and ART biosynthetic pathways. - Increased GST size (+49%) and density (+38%).	-	High
Non-*Artemisia* plants	Guo et al., [40]	2025	Transient expression in *Nicotiana benthamiana* with ART pathway genes + *DHAADH*	ART detected at 0.041 mg g^−1^ DW after UV treatment; DHAA produced at 0.51 mg g^−1^ DW	- Reconstruction of ART pathway in tobacco possible. - UV light promotes DHAA auto-oxidation to ART	Low ART yield in plant system; requires external trigger for ART formation	High
Non-*Artemisia* plants	Firsov et al., [41]	2021	Heterologous Biosynthesis in *Chrysanthemum morifolium* (with *A. annua* genes *mtADS*, *CYP71AV1*, *CPR*, *DBR2* and yeast *tHMGR*)	ART was not detected via GC-MS.	The study confirmed the feasibility of transplanting an active ART biosynthetic pathway into *chrysanthemum*.	The lack of ART accumulation suggests that host biochemical backgrounds limit production efficacy.	High
Non-*Artemisia* plants	Randrianarivo et al., [42]	2021	Extraction from an atypical plant source	Isolation of a new stereoisomer, (−)-6-epi-artemisinin, from *Saldinia proboscidea*.	The isomer displayed comparable bioactivities to (+)-artemisinin in antimalarial and antiproliferative assays, indicating that the change in configuration is not critical to biological properties.	Only 2.5 mg of the compound was obtained from 490 g of dried plant powder.	High
Non-*Artemisia* plants	Firsov et al., [43]	2020	Metabolic engineering of *Chrysanthemum morifolium* using binary vectors to introduce ART biosynthesis genes.	- Confirmation of transcription of all five genes in two specific lines. - Qualitative detection of ART presence via thin layer chromatography.	- It is feasible to transfer a genetic module encoding the entire biochemical pathway into the *chrysanthemum* genome. -Targeting the *ADS* gene to the mitochondria resulted in transcription of all genes.	- As low transformation efficiency, the frequency of obtaining lines with all target genes was very low (0.33%). - as a metabolic gap, while ART was detected, its precursor was not detected.	High
Semi-synthetic routes	Guo et al., [40]	2025	Metabolic engineering of *S. cerevisiae* with optimized DHAADH for DHAA production	3.97 g L^−1^ of DHAA in a 5 L bioreactor; 22.67 g L^−1^ of artemisinic acid (AA) in same system	Discovery of DHAADH catalyzing bidirectional AA↔DHAA conversion; DHAA can auto-oxidize to ART	Terminal pathway of ART remains unclear; direct ART production in heterologous systems not yet achieved	High
Semi-synthetic routes	Chen et al., [44]	2023	Semi-synthesis via “Dark” Singlet Oxygen: Chemical conversion of DHAA using molybdate-catalyzed (Na_2_MoO_4_) of H_2_O_2_.	Achieved a 41% yield of ART on a 3 g scale (producing 1.48 g of pure product).	The method provides a scalable and cost-effective synthesis using open flask at ambient temperature, eliminating the need for photochemical equipment, or complex continuous-flow reactors.	The overall reaction time is relatively long (12–14 h for the first step and 2 days for the second step) compared to photo-irradiation based approaches.	High

**Table 2:** Comparative Analysis of Alternative ART Production Platforms.

Production Platform	Key Yield/Performance Metrics	Scalability and Cost Implications	TRL *	Key Advantages	Key Limitations
ART enhancement in *A. annua*	*A. annua* baseline: 0.01–1.5% DW Yield increase up to 26 mg g^−1^ DW	High Scalability Low Cost	9 (Commercial)	Natural source	Variable yields; Success de-pends on environmental interaction
In vitro culture (callus/suspension)	Yield up to 25 mg g^−1^ DW	Medium/High Cost: Requires bioreactors; trade-offs between biomass and ART	5–6 (Pilot)	GMP compliance; controlled environment.	Lower ART than control plants; optimization challenges.
Heterologous expression (non-*Artemisia* plants)	Low ART yield (e.g., 0.041 mg g^−1^ DW in *N. benthamiana*).	Low Scalability: Currently limited to laboratory scale.	4 (Lab Validation)	Potential for novel plant chassis.	Low yields; ART often not detected or requires external triggers (UV).
Semi-Synthetic (Yeast/Chemical)	DHAA yield: 3.97 g L^−1^	High scalability; Decouples supply from agriculture.	3–4	Consistent supply; rapid production cycle.	High startup costs

Abbreviation: * TRL, Tech. Readiness Level.

## Data Availability

No new data were created or analyzed in this study. Data sharing is not applicable to this article.

## Abbreviations

The following abbreviations are used in this manuscript:

ABA	Abscisic acid
ACT	Artemisinin-based Combination Therapies
ADS	Amorpha-4,11-diene synthase
ALDH1	Aldehyde dehydrogenase 1
ART	Artemisinin
BFS	β-farnesene synthase
CPS	β-caryophyllene synthase
CYP	Cytochrome P 450 CYP71AV1
DBR2	Double bond reductase 2
DHAA	Dihydroartemisinic acid
DL	Deep learning
DW	Dry Weight
DXR	1-deoxyxylulouse 5-phosphate reductoisomerase
DXS	1-deoxyxylulose 5-phosphate synthase
FPP	Farnesyl pyrophosphate
FPS	Farnesyl pyrophosphate synthase
GA	Gibberellin
GST	Glandular secretory trichomes
HMGR	3-hydroxy-3-methylglutaryl-CoA reductase
HMGS	3-hydroxy-3-methylglutaryl-CoA synthase
IAA	Indole-3-acetic acid
JA	Jasmonic acid
MeJA	Methyl jasmonate
ML	Machine learning
SA	Salicylic acid
SL	Strigolactone
TF	Transcription factor
